# Physical Delithiation of Epitaxial LiCoO_2_ Battery Cathodes as a Platform for Surface Electronic Structure Investigation

**DOI:** 10.1021/acsami.3c06147

**Published:** 2023-07-19

**Authors:** Elena Salagre, Pilar Segovia, Miguel Ángel González-Barrio, Matteo Jugovac, Paolo Moras, Igor Pis, Federica Bondino, Justin Pearson, Richmond Shiwei Wang, Ichiro Takeuchi, Elliot J. Fuller, Alec A. Talin, Arantzazu Mascaraque, Enrique G. Michel

**Affiliations:** † Dto. Física Materia Condensada, Univ. Autónoma de Madrid, 28049 Madrid, Spain; ‡ IFIMAC (Condensed Matter Physics Center), Univ. Autónoma de Madrid, 28049 Madrid, Spain; § Dto. Física de Materiales, Fac. Ciencias Físicas, Univ. Complutense de Madrid, 28040 Madrid, Spain; ∥ Istituto di Struttura della Materia-CNR (ISM-CNR), Trieste 34149, Italy; ⊥ IOM CNR Laboratorio TASC, AREA Science Park, Trieste 34149, Italy; # Materials Science and Engineering, Univ. of Maryland, College Park, Maryland 20742, United States; ∇ Sandia National Laboratories, 7011 East Avenue, Livermore, California 94550, United States

**Keywords:** lithium cobalt oxide, lithium ion batteries, photoemission spectroscopy, sputtering, absorption spectroscopy

## Abstract

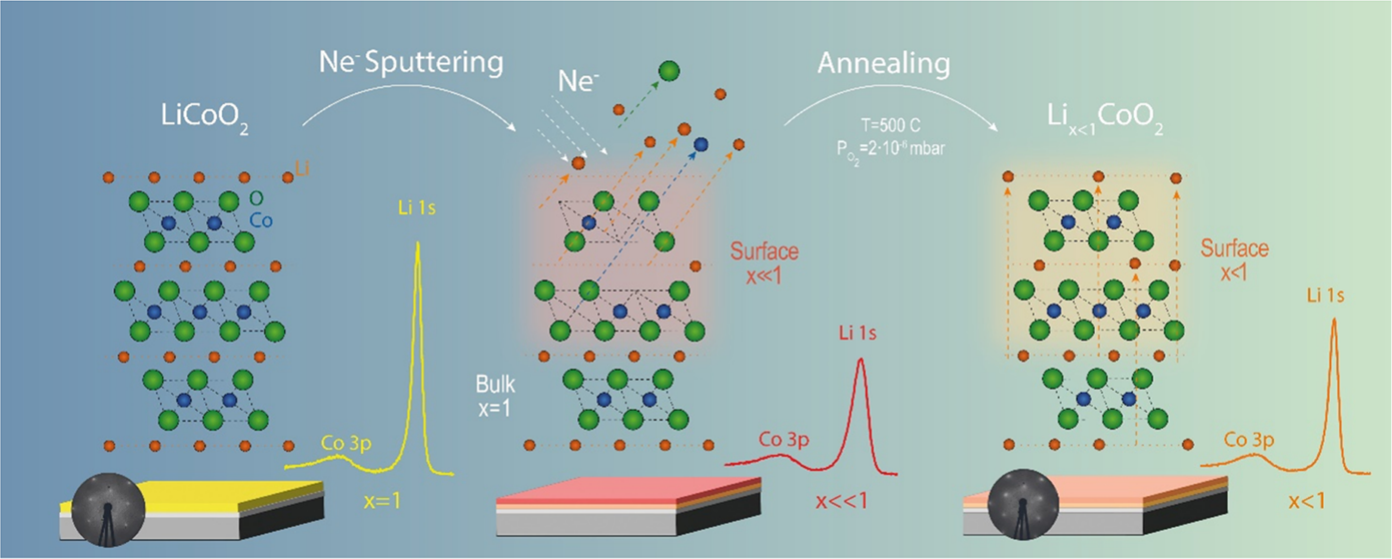

We report a novel delithiation process for epitaxial thin films of LiCoO_2_(001) cathodes using only physical methods, based on ion sputtering and annealing cycles. Preferential Li sputtering followed by annealing produces a surface layer with a Li molar fraction in the range 0.5 < *x* < 1, characterized by good crystalline quality. This delithiation procedure allows the unambiguous identification of the effects of Li extraction without chemical byproducts and experimental complications caused by electrolyte interaction with the LiCoO_2_ surface. An analysis by X-ray photoelectron spectroscopy (XPS) and X-ray absorption spectroscopy (XAS) provides a detailed description of the delithiation process and the role of O and Co atoms in charge compensation. We observe the simultaneous formation of Co^4+^ ions and of holes localized near O atoms upon Li removal, while the surface shows a (2 × 1) reconstruction. The delithiation method described here can be applied to other crystalline battery elements and provide information on their properties that is otherwise difficult to obtain.

## Introduction

Understanding interfacial stability in batteries and the reasons for degradation over time is critical to the development of many important applications. For example, all-solid-state Li-ion batteries are a cutting-edge technology that can achieve the levels of safety and energy density required in many applications.^1^ However, despite high expectations, all-solid-state Li-ion batteries have still not achieved extensive commercial use because their power density and capacity retention are strongly limited by the quality of the interfaces between components.^2−4^ Our understanding of the critical role of the interface in the various mechanisms leading to instability over time is still too limited, in part due to the complex interactions^5^ between anode and cathode interfaces with solid-state electrolytes, as highlighted by recent studies using crystallographically oriented and faceted cathodes.^5−8^ The evolution of the interface profoundly affects the interaction between the cathode and the electrolytes, which include both solid and liquid forms. These intricate interfacial dynamics are critical to battery performance, so understanding the surface in detail is relevant to improving battery operation.^2,9^ Changes in the voltammograms between oriented and amorphous samples highlight the importance of the surface and its orientation in applications such as catalysis.^8,10^

Improving the performance of a battery element (anode, cathode, electrolyte) depends on knowledge of its structure and physicochemical properties. Surface spectroscopy and structural characterization methods are powerful tools to provide a mechanistic view of the above processes, but the presence of electrolytes, solid or liquid, during the delithiation process greatly complicates this experimental approach and, above all, the interpretation of the results.^11,12^ To overcome this problem, the development of new advanced characterization strategies is of paramount importance.

Li*_x_*CoO2 (LCO) is a relevant example. It has been extensively used as a battery cathode since 1991,^13,14^ due to its high-energy capacity and long cycle life. More recently, it has also emerged as an attractive material for electrochemical random access memory (ECRAM), based on electronic resistance switching controlled by Li-ion intercalation.^15−17^ However, our knowledge of the electronic band structure of LCO and the changes it undergoes after delithiation is limited to a few studies, due to the difficulty in obtaining delithiated LCO crystalline films of sufficient quality.^18−20^ LCO crystallizes in a rhombohedral structure, space group *R*3̅*m* (166),^21^ which can accommodate a wide-ranging Li^22^ concentration. This property is related to both the high mobility of the Li atoms and the ability of the lattice to stabilize different Li molar fractions. LiCoO_2_ is a band insulator (α phase, described above), but Li*_x_*CoO_2_ is metallic for 0.5 < *x* < 0.75 (β phase, with a structure similar to that of the α phase).^23^ For 0.75 < *x* < 0.94, Li*_x_*CoO_2_ is also metallic, but the α and β phases coexist. This range coincides with the Li content most commonly used when LCO acts as a battery cathode. For *x* > 0.94, Li*_x_*CoO_2_ is insulating and it is debated whether the insulator–metal transition at *x* = 0.94 is of the Mott^24−26^ or Anderson^27,28^ type. Around *x* = 0.5 (0.46 < *x* < 0.54), a monoclinic phase (*P*2/*m* group)^29,30^ causes irreversible changes and can lead to structural damage.^31^ The Li deficiency induces a wide range of phenomena, including order/disorder transitions,^32^ oxygen sliding, and the insulator–metal transition at *x* = 0.94.^24,27^ The nature of the charge compensation that occurs when Li is removed is controversial, but it is a relevant question given the prospect of LCO as a memory device.^15,16^ It is also relevant to interpret the behavior of the cathode conductivity, which plays an important role in battery performance.^8,33^

Here, we report on a novel and efficient approach to achieve delithiation of an epitaxial, crystalline cathode, without the need to use a liquid or solid electrolyte. The new method is based on the physical removal of Li ions by Ne sputtering. In contrast to electrochemical or purely chemical delithiation, which either generate a solid electrolyte interphase (SEI) that alters the material properties^34^ or can strongly affect the crystalline structure,^31,35^ the physical delithiation method described here can be applied to an epitaxial LCO cathode without significant loss of crystalline quality for delithiation ranges up to *x* ≈ 0.5. Physical delithiation can also shed light on the analysis of interface formation with a solid electrolyte, as it provides information on the chemical state of the cathode and therefore on possible reactions with the electrolyte at the different stages of battery cycling, without having to infer the origin of the interface layers by changing the electrolyte chemistry.^36^ The effects of delithiation are monitored using X-ray photoelectron spectroscopy (XPS) and X-ray absorption spectroscopy (XAS) and include the identification of the mechanism of charge compensation that takes place at Co atoms (change from Co^3+^ to Co^4+^) and simultaneously at O atoms (observation of holes). We find that the stoichiometric LCO(001) surface presents a (2 × 1) reconstruction, which is maintained for a certain delithiation range. We identify specific surface components in Li 1s and O 1s and interpret the surface reconstruction as being due to 0.5 monolayer (ML) of ordered Li atoms. The delithiation strategy found is applicable to solid-state cathodes and electrolytes, and it can also be used to grow interfaces between them with specific properties, opening a way to obtain new information about the evolution of their properties with Li content.

## Experimental Section

The samples used in this study were 200–400 nm thick high-quality epitaxial films of LiCoO_2_(001) grown by pulsed laser deposition (PLD) on Nb-SrTiO_3_(111) covered with a buffer layer of SrRuO_3_(111). The buffer layer is required to provide electrical contact to LiCoO_2_.^37^ LiCoO_2_(104) epitaxial films grown on SrTiO_3_(100) are also used for comparison. Their quality was checked with X-ray diffraction (XRD), Raman spectroscopy, and atomic force microscopy (AFM). Once in ultrahigh vacuum, the samples are degassed at 300 °C for several hours. Afterward, the samples undergo several cleaning cycles that involve annealing in oxygen (10^–6^ mbar) at 550 °C for 30 min followed by a 5 min annealing in ultrahigh vacuum (UHV) to 430 °C and slow cooling (40 °C/min) down to room temperature. This multistep process removes the surface contamination without affecting the crystalline quality or stoichiometry. We expect that the annealing process favors the formation of a stable surface termination, corresponding to the equilibrium surface structure under the described annealing conditions.

The surface crystalline quality was checked using low-energy electron diffraction (LEED). The chemical composition of the samples was analyzed with XPS, at various photon energies, in a UHV chamber (pressure < 2 × 10^–10^ mbar) located at the VUV-Photoemission beamline at the Elettra storage ring in Trieste (Italy). Core-level peaks were monitored using a Scienta R4000 electron analyzer. The measurements were performed at room temperature. XAS and XPS measurements were acquired at the BACH beamline of the Elettra storage ring. XAS is performed using total electron yield by measuring the drain current through the sample using a picoammeter and a Scienta R3000 electron analyzer is used for the XPS measurements. XAS was used to measure the Co L and O K edges as a function of Li molar fraction. The quality and cleanliness of the surface were checked by measuring overview XPS spectra taken with *h*ν = 670 eV and monitoring the C 1s and O 1s peaks during the cleaning process (Figure SI 2). The Li molar fraction is determined from the Co 3p and Li 1s ratio and is carefully monitored during the cleaning process to prevent any undesired delithiation. The line shape of the core levels was fitted using a Shirley background and asymmetric pseudo-Voigt functions. The fit is optimized using a Levenberg–Marquardt algorithm with a routine running in Igor Pro (WaveMatrix, Inc.).^38^ The quality of the fit is judged from a reliability factor, the normalized χ^2^.

## Results and Discussion

### Physical Delithiation

**Figure 1:**
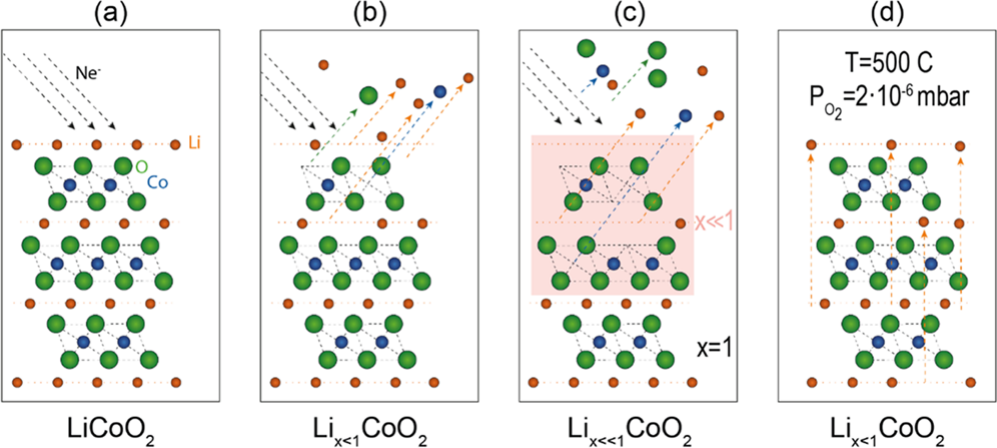
(a–d) Schemes of the delithiation process using Ne^+^ sputtering and annealing. Ne^+^ sputtering (a) induces preferential Li removal (b) in the ion penetration layer; some damage to the atomic structure is also produced due to the removal of Co and O atoms. (c) After annealing in O_2_, the penetration layer recovers its crystalline quality, but it remains depleted in Li (d).

Pristine LCO epitaxial films are partially delithiated through Ne^+^ sputtering under controlled conditions. The ion energy was selected based on previous simulation work of the interaction of Ne^+^ with an LCO target performed with the software package SRIM^39^ (see Figure SI.3). Li atoms are removed more efficiently than the heavier Co or O atoms. The use of Ne^+^ instead of the heavier Ar^+^ provides also a larger fraction of Li atoms removed (see Figure SI.3). Different Ne^+^ energies and times were used to optimize the outcome in each sample and for different delithiation degrees. The initial sputtering time and fluence are selected considering the known performance of the apparatus. Typical values are 600 eV energy and sputtering times of less than 5 minutes with a sample current of 30 μA corresponding to a ion flux of 7.7 × 10^14^ e/s cm^2^. The samples are annealed at 500 C in 1 × 10^–6^ mbar O_2_ in order to facilitate the crystal reordering and to minimize any possible oxygen deficiency. After sputtering, the surface Li content is dramatically reduced, but this Li concentration is partially recovered after annealing. Our approach and the atomic processes are shown schematically in Figure 1. The thickness of the layer affected by the sputtering (delithiated layer) depends on the sputtering energy used, but generally is limited to the first few atomic layers. An estimate of the affected thickness at 600 eV sputtering energy is 5 nm. (see Figure SI 3). Considering the sample area affected by a standard ion gun, a delithiated thickness of several nanometers for samples of ∼5 cm^2^ is easily reached in a few minutes. A thicker layer can be delithiated using beam energies higher than 600 eV.

**Figure 2:**
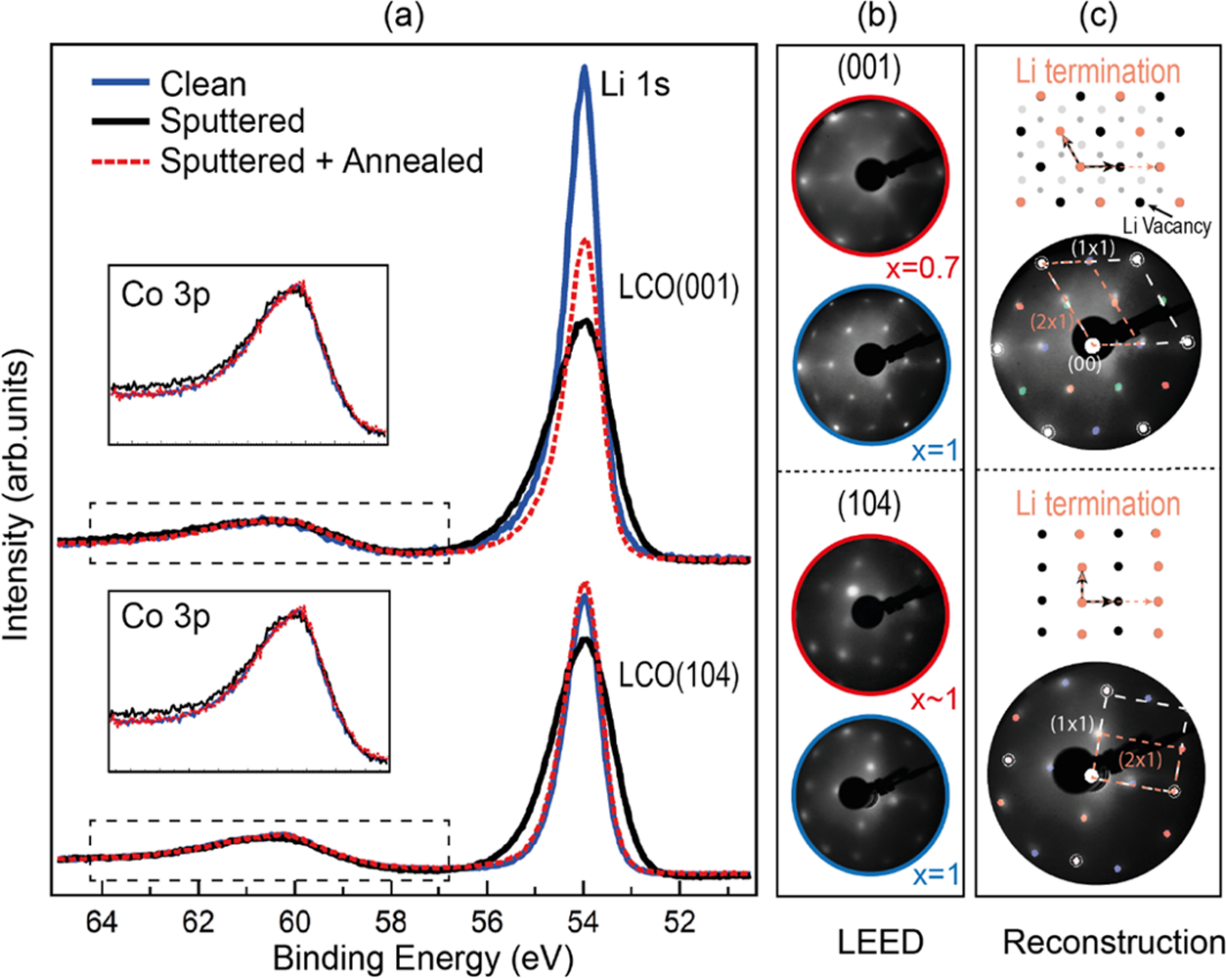
(a) Co 3p and Li 1s XPS for *h*v = 150 eV, corresponding to the pristine/stoichiometric (blue), Ne^+^ sputtered (black) and sputtered and annealed in O_2_ (red) samples. The spectra are normalized to the intensity of Co 3p. The top row corresponds to samples of (001) orientation, and the bottom row corresponds to (104) orientation. LEED patterns are shown in (b) for the pristine and sputtered-annealed samples. All LEED patterns are taken at *E* = 95 eV. Note that both surfaces are reconstructed. (c) Scheme of the LEED patterns highlighting the (1 × 1) reciprocal cell (white) and the (2 × 1) domains (colored), together with simple schemes of the real space.

Ne^+^ sputtering depletes the Li concentration in the penetration range of ions, which corresponds to the top crystalline layers. A gradient of Li concentration is built up between the surface delithiated layers and the stoichiometric bulk. As the bulk acts as a Li reservoir during the annealing process, part of the Li removed from the surface is recovered after annealing due to diffusion from the bulk. In the case of LCO(104), the stoichiometric Li molar fraction is almost completely recovered after annealing. In turn, in the case of LCO(001), the surface layers remain delithiated after annealing (Figure 2). The reason of this difference is that Li diffusion along the (001) direction is significantly lower than along any other crystalline direction because Li atoms have to overcome the diffusion barrier formed by the CoO_2_ layers.^8,29^ In the case of (104) oriented epitaxial films, Li crystalline planes are not parallel to the surface and Li diffusion toward the surface is very easy. A Li-depleted layer is not observed in this case (see Figures 2 and SI 1). Therefore, we propose that poor diffusion along (001) is responsible for the observed stabilization of a Li-depleted layer at the surface of a (001) oriented epitaxial film. By this method, the Li molar fraction can be tuned to any desired value in the 0.5 < *x* < 1 range. The crystalline quality of the delithiated sample is validated using LEED (Figure 2), which shows sharp diffraction spots for delithiations in the 0.5 < *x* < 1 range. A stable structure is formed in this delithiation range. Figure 2 summarizes the delithiation process through the changes observed in the Co 3p-Li 1s region. The XPS spectra are normalized to the intensity of Co 3p to permit an easy comparison between them. After Ne^+^ sputtering (black line), the (001) film (top row) presents a lower Li 1s relative intensity than the pristine sample (blue line), and Co 3p presents a higher intensity on the high binding energy (BE) side (see insets in Figure 2), denoting the presence of Co atoms with a different oxidation state, related to the local disorder. After annealing (red line) the line shapes are completely recovered, but a smaller amount of Li is present. The annealing process induces some increase in the Li concentration also in the case of (001) orientation because of the growth mode of the epitaxial film, which is composed of domains that expose a certain fraction of {104} planes with a high Li-ion diffusivity.^37^ Given this mechanism, we expect that the physical method of deintercalation can be extended to other materials with layered structures and with a significant difference in the diffusion coefficient along relevant crystalline directions.

### Characterization of LCO during the Delithiation Process

**Figure 3:**
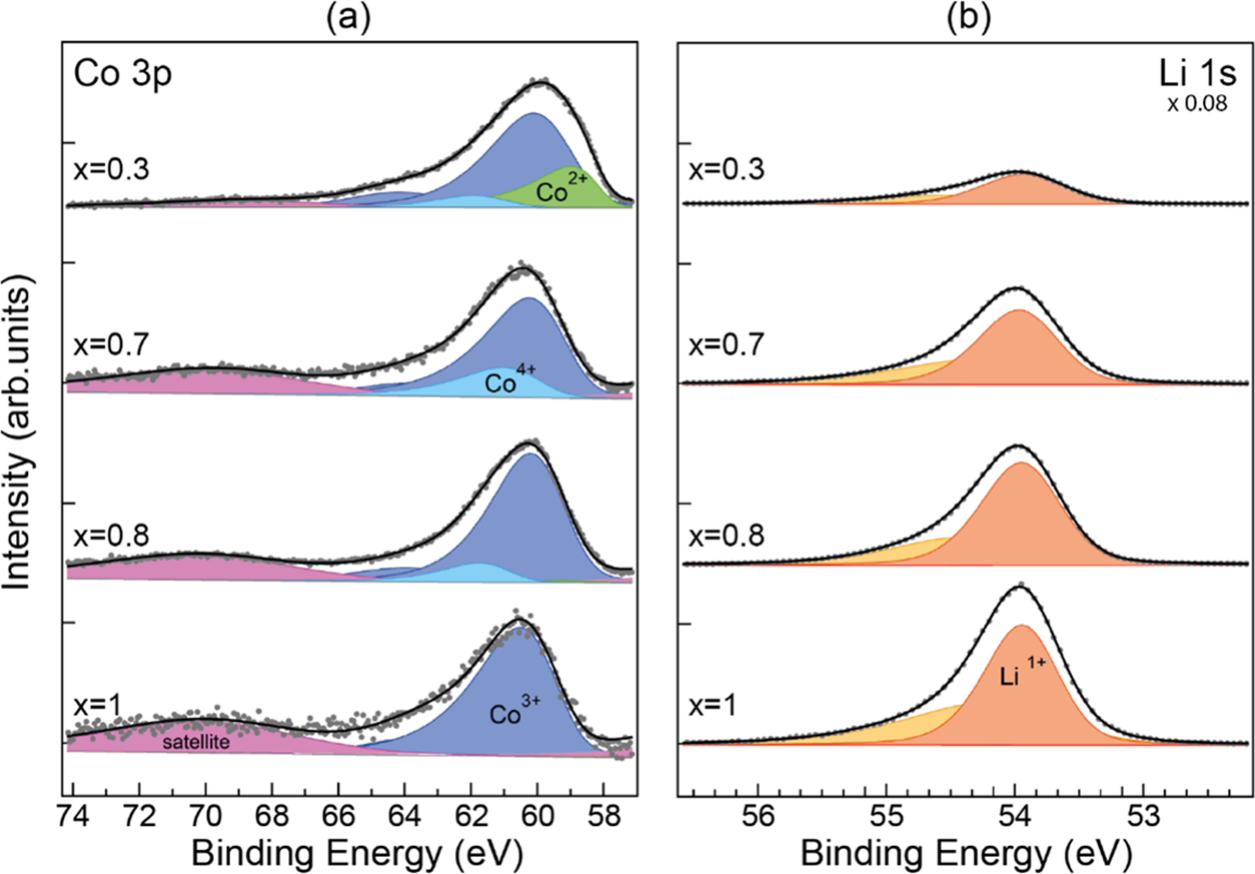
XPS spectra of Co 3p (a) and Li 1s (b) for a pristine sample (*x* = 1) and several delithiated samples (Li molar fractions *x* = 0.8, 0.7, and 0.3) highlighting the deconvolution of the peaks, *h*v = 150 eV. In both graphs, the intensity is normalized to the Co 3p peak to observe the decrease in Li ratio. Li 1s has been scaled by 0.08 for comparison with Co 3p.

We monitored O 1s, Co 2p, Li 1s, and Co 3p core levels and O K and Co L_2,3_ absorption edges during the delithiation sequence. Figures 2 and 3 demonstrate the efficiency of the method. The relevant core levels provide information about the stoichiometry and oxidation state of the different elements present in the sample. However, the analysis is complex in the case of transition-metal oxides, and in particular in the case of LCO. On the one hand, the crystal field, due to the intrinsic symmetries of the structure (octahedral symmetry around Co atoms), modifies the splitting^40^ and filling of valence band states.^41,42^ On the other hand, Co core levels present a well-known multiplet splitting that gives rise to satellites.^43^ This complicates the interpretation of the XPS data. The analysis has to be done by combining information from XAS in order to explain the behavior of all species present and their chemical state.

### Li 1s Core Level

**Figure 4:**
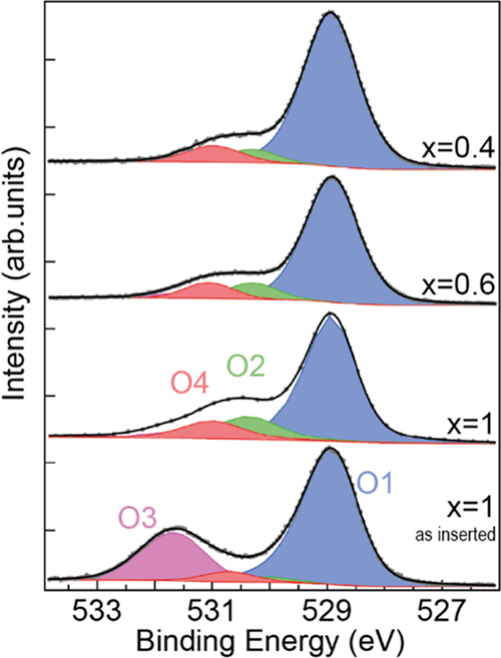
XPS spectra of O 1s (*h*v = 670 eV) for different preparation conditions and core-level deconvolution as a function of Li molar fraction (*x*). Oxygen components are O1 (main LCO component), O2 and O4 (LCO surface), and O3 (surface contamination).

Our results of the Li 1s peak shape indicate surface termination of the LCO crystal by a Li layer. The Li 1s core level in LCO has a reported BE of ∼54 eV.^44−47^ The BE of Li 1s is not sensitive to the Li molar fraction, as the oxidation state of Li is always +1. However, the line shape of Li 1s depends on the value of the Li molar fraction, as shown in Figure 3b. In particular, there is a clear asymmetry of the high binding energy side of Li 1s (see also Figure SI.4). Metallic samples usually present an asymmetric core level. As there is a range of phase coexistence for 0.75 < *x* < 0.94, we would expect an increase in the asymmetry as the Li molar fraction decreases in this range. However, the asymmetry does not increase for *x* > 0.75. Thus, we can rule out this explanation and attribute the asymmetry to an additional Li 1s component. Li 1s is deconvoluted in Figure 3 using an additional component at ∼54.6 eV BE. Previous studies^48,49^ regarding Li compounds associate a Li 1s asymmetry with the possible presence of Li_2_O. In this case, there would also be a corresponding O 1s component at 528.5 eV and spectral signatures from Li_2_O in the valence band.^50^ As no Li_2_O O 1s component (see Figure 4) or other features in the valence band related to Li_2_O (not shown) are observed, we also discard this explanation. Finally, the additional Li 1s component could be related to the surface termination, and then it would be present in the sample from the beginning. The ratio of the additional component and the main peak is approximately constant (see Figure SI.5) as the delithiation progresses, or at most, the high BE component decreases slightly, in agreement with this interpretation. For *h*ν = 150 eV, when the Inelastic Mean Free Path (IMFP) is around 5 Å, the last Li layer has a 60% contribution to the total Li signal from the first 4 Li atomic planes. In agreement with this explanation, at *h*ν = 670 eV, the probing depth is larger, with an IMFP of 14 Å, and the last Li layer has less relative importance (<20%). This effect reduces the asymmetry at *h*ν = 670 eV, as found in Figure SI.4. All of these observations support a surface component as the explanation for the Li 1s asymmetry, which we attribute to the surface termination of the LCO crystal by a Li layer.

### Co Core Levels

Some authors^45,51,52^ propose that delithiation is related to a valence change of Co, from Co^3+^ in the stoichiometric LCO structure to Co^4+^ in Li*_x_*CoO_2_ (0.5 < *x* < 1). This change would compensate for the charge lost after delithiation. In turn, other authors^28,53−56^ point to the involvement of oxygen atoms in the process so that charge compensation would take place by the formation of holes at oxygen atoms, whereas Co atoms would maintain a nominal oxidation state of 3+. Some works consider that both mechanisms operate in LCO.^20,44,57,58^ Information on the origin of charge compensation is important to have a full understanding of the electronic structure of the cathode as a function of the Li content, which is a relevant question in view of the prospect of LCO as a memory device.

Figure 3a shows the Co 3p core level measured with *h*ν = 150 eV. The data for both panels are normalized to the intensity of the Co 3p peak. Note the relative decrease of the Li 1s peak as the delithiation process proceeds. The Li/Co ratio is calculated following a deconvolution of the line shape and a comparison of the Co 3p and Li 1s intensities, which provides the *x* value (Li molar fraction).

The deconvolution of the Co 3p line shape provides information on the delithiation process. For the pristine sample, there is a main component (dark blue) corresponding to Co^3+^ in the LCO lattice and a broad satellite (pink)^43,52^ related to multiplet splitting. The satellite BE appears shifted by 9.6 eV, in agreement with previous data for Co^3+^.^45,59^^45,59^ When Li is removed, the Co 3p peak broadens and a new component appears at higher binding energy (light blue). This component grows for smaller Li molar fractions. We assign this component to Co^4+^, which is the expected oxidation state of Co near a Li vacancy. The BE of the Co^4+^ component is in good agreement with previous findings.^44,59^ The relative intensity of Co^3+^ and of its satellite decrease as the delithiation proceeds. For large delithiation values (*x* < 0.4), the line shape broadens on the low binding energy side, indicating the appearance of a new component (green), which is identified with Co^2+^. The appearance of Co^2+^ is related to the formation of oxygen vacancies, as established in previous work.^60^ The observation of Co^2+^ for high delithiation (*x* < 0.4) is related to lattice damage in this delithiation range due to the appearance of the monoclinic phase below *x* = 0.45, which leads to irreversible damage.^45,46^

We have focused so far on Co 3p since it is advantageous for determining the Li molar fraction due to its proximity to Li 1s. Additional analysis was simultaneously done on Co 2p (see Figure SI.6), which is the standard Co reference. Co 2p spectra for *x* = 1 show a satellite change consistent with the presence of Co^3+^ only (S3). The broadening of the Co 2p_3/2_ component as delithiation proceeds is consistent with the formation of Co^4+^.^44,59^ The satellite S4 is related to Co^2+^. It is observed for *x* ≤ 0.5 as expected from structural damage in the highly delithiated regime. The overall line shape of Co 2p has a complex interpretation, where the identification of components based on their shift and valence is not straightforward.^42^

### O 1s Core Level

Figure 4 shows the O 1s line shape changes during sample cleaning and delithiation. Contrary to previous observations in studies using chemical delithiation, no additional component from interaction with acids exists in this case. Component O3, probably related to hydroxylation, is observed in the sample before cleaning. It disappears after preparing a clean and well-ordered surface. The main O1 component is attributed to O atoms in the LCO structure. Two additional components, O2 and O4, appear on the clean, well-ordered surface and correspond to less reduced oxygen atoms (higher BE). Any surface oxygen atoms will have a lower coordination and appear in a less reduced chemical state, closer to atomic oxygen, while oxygen atoms in the bulk remain fully reduced. Once the sample is clean, the intensity ratio between components remains approximately constant, although a small decrease in the ratio O2/O4 is observed as the delithiation proceeds. This minor change is attributed to a decrease in the surface quality (e.g., by an increase of roughness) after subsequent sputtering and annealing cycles.

### Surface Termination

LCO(001) is a polar surface (Type II in the Tasker classification^61^) which introduces additional complications in the analysis of its properties. The crystal contains charged alternating layers of *q* = +1 (Li layers) and *q* = −1 (O–Co–O layers). In order to keep overall charge neutrality, the charge must be redistributed at the surface termination. The possibilities found by the theoretical work in refs (62) and (63) are termination by 0.5 ML of Li, O termination together with mixed valence in Co–O planes, oxygen deficiency (vacancies), or the presence of delocalized charge. Out of these possibilities, only a Li surface termination is compatible with our experimental results for the following reasons. First, the stoichiometric LiCoO_2_ surface does not present a mixture of Co^3+^ and Co^4+^, as observed for delithiated surfaces, in view of the Co 2p and Co 3p core-level line shape and deconvolution. Second, O vacancies are related to the presence of Co^2+^, which is not observed for Li molar fractions above 0.5. Finally, the presence of delocalized charge is not compatible with the insulating character of the surface, as detected by valence band photoemission. We show the valence band of the stoichiometric LCO surface in Figure SI.8 in the Supporting Information. We conclude that the surface is terminated by 0.5 ML Li. The higher BE of the Li 1s component is explained by a lower crystal potential and not due to a lower charge.^64^ The presence of the 0.5 Li termination is also supported by the observation of a LEED pattern corresponding either to a (2 × 2) or to a three-domain (2 × 1) reconstruction, as expected for a well-ordered layer. Previous STM studies support the Li termination of the surface,^19^ also favored in other studies.^64^

It is worth mentioning that the LEED pattern does not discern between a (2 × 2) or a three-domain (2 × 1), and that the domains are not directly observed since they are expected to be much smaller than the transfer width of the LEED instrument. We favor the three-domain (2 × 1) possibility as it is easily compatible with a 0.5 ML coverage (see Figure SI.7). Note that these reconstructions can be very sensitive to the sample preparation, as was observed for other materials.^65,66^

The O 1s core level presents also two surface components O2 and O4 at higher BE indicating a less negative charge,^46^ i.e., oxygen atoms less reduced than in bulk LCO. The two O 1s surface components are attributed to surface oxygen atoms in different configurations in the surface termination layer.^63^

### X-ray Absorption Edges

**Figure 5:**
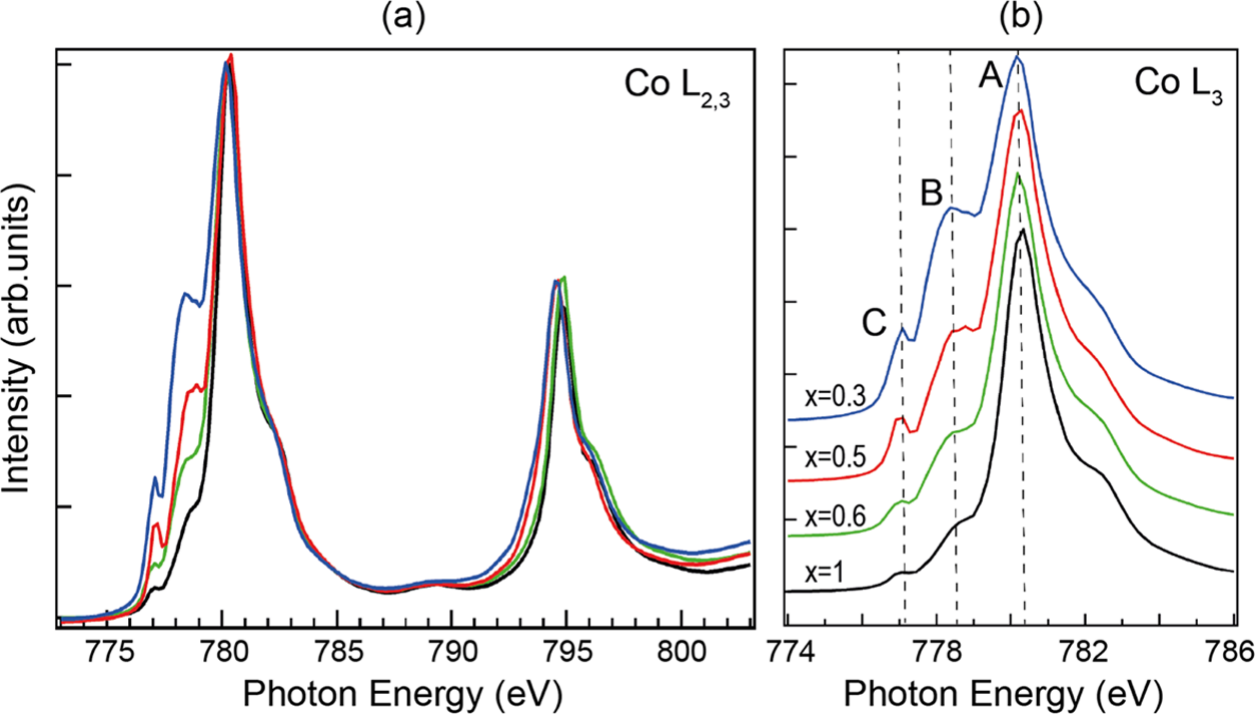
(a) XAS spectra of Co L_3,2_-edge for different Li molar fractions taken at an incidence angle of 30°, with horizontal linear polarization (no changes for vertical polarization observed or after changing the incidence angle). (b) Close-up view of the L_3_-edge. The line color denotes the Li molar fraction: black *x* = 1, green *x* = 0.6, red *x* = 0.5, blue *x* = 0.3.

The Co L-edge and O K-edge were monitored using x-ray absorption spectroscopy (XAS) as a function of Li molar fraction. The delithiation process removes charge from the valence band and generates empty states. New transitions, which appear as prepeaks in the absorption edges, are present in the delithiated sample. Figure 5 shows the Co L-edge and the appearance of two peaks that grow as the Li molar fraction decreases when the delithiation proceeds (labeled as B, at 1.7 eV from the main peak A, and C, at 3.2 eV from A, in Figure 5b).

The reported Co L-edge for stoichiometric LCO has a line shape very similar to the edge shown in Figure 5a for the *x* = 1 case,^20−22^ with two prepeaks (labeled here B and C). However, the Co L-edge of single-crystalline LCO with different degrees of delithiation presents only one peak in the position of B,^20,67^ which is attributed to the formation of Co^4+^. On the other hand, the Co L-edge corresponding to Co^2+^ presents two prepeaks near C and B (the latter formed by two structures). We note that the intensity ratio between B and C is not maintained during delithiation. For *x* = 1, B takes 10% of the L_3_, while for *x* = 0.5, it reaches almost 20%. Meanwhile, component C corresponds to less than 1% of L_3_ for the stoichiometric case and around 3% for the *x* = 0.5 case. The internal structure of B also suffers a clear change. We conclude that B has an underlying component related to Co^4+^, which is significant for low delithiations, while a second component related to Co^2+^ in a very near location grows for larger delithiations (*x* ≤ 0.5). This interpretation is in agreement with the observation with XPS of Co^2+^ formation for large delithiation (*x* ≤ 0.5, Figures 3 and SI.6). The fact that a small amount of Co^2+^ is observed with XAS in the stoichiometric sample can be attributed to defects during growth or preparation that are not visible with XPS, due to its smaller probing depth.

**Figure 6:**
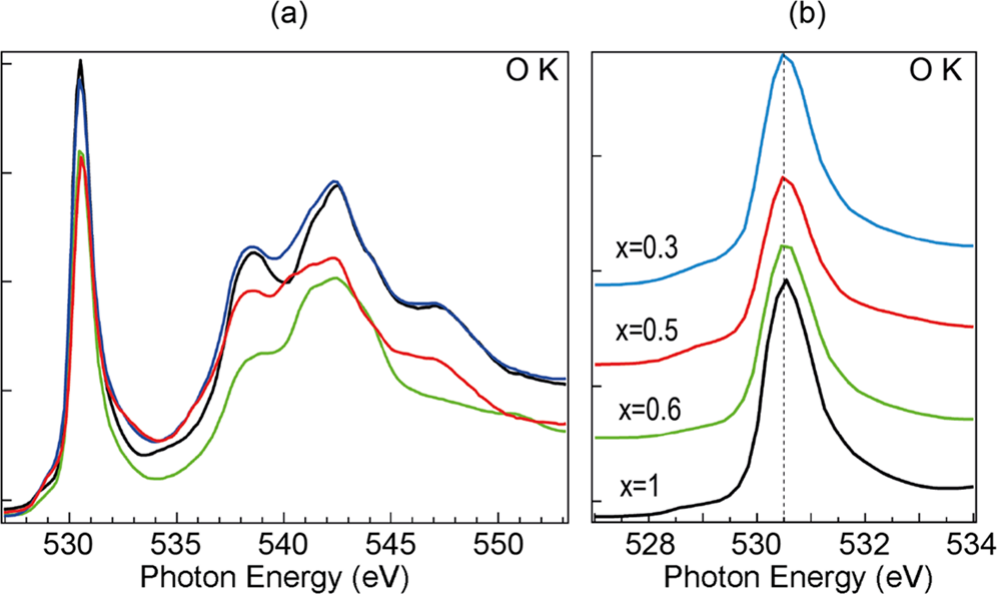
(a) XAS spectra of O K-edge corresponding to different Li molar fractions taken at an incidence angle of 30° with horizontal polarization (no change for vertical polarization observed). (b) Close-up view of the main peak at low energy. The line color denotes the Li molar fraction: black *x* = 1, green *x* = 0.6, red *x* = 0.5, blue *x* = 0.3.

Figure 6 shows the O K-edge as a function of Li molar fraction as the delithiation progresses. The O K absorption edge is dominated by a prominent peak at 530.5 eV that corresponds to the transition of O 1s electrons into Co 3d hybridized with O 2pσ orbitals (e_g_*).^55^ One broad prepeak grows near 529 eV as the delithiation proceeds, indicating the appearance and strengthening of new transitions as the Li molar fraction decreases. Delithiation also induces changes in the 535–550 eV region, corresponding to hybridization of O 2p and Co 4sp states.^68^

The simultaneous changes of Co L and O K edges with the appearance of prepeaks indicate that charge compensation upon Li deintercalation takes place at Co atoms, but holes at O atoms are also observed.

Charge compensation in LCO upon delithiation has been a controversial topic.^54,57^ We observe charge compensation in Co atoms, with the appearance of Co^4+^, as evidenced by the changes in Co 3p core level and in the Co L-edge absorption spectra. In addition, we also observe changes in the O K-edge that suggest the existence of oxygen holes, indicating that O atoms also play a role in charge compensation. The discrepancies found in the literature can be attributed, among other factors, to the starting samples (which present very clearly different Li molar fractions), but also to the delithiation method. The physical (sputtering + annealing) method used in this work results in a gradient of Li concentration along the surface normal, being the surface itself mostly affected by Ne^+^ ions. The annealing process compensates in part for this inhomogeneity, and our surface-sensitive techniques minimize the effect of this gradient in the obtained data and conclusions, but it certainly generates a Li distribution that may be different from the distribution found using electrochemical cycling.^57^

## Conclusions

We demonstrate that sputtering of LCO(001) films with Ne^+^ ions delithiates the top few layers corresponding to the ion penetration depth, followed by a partial recovery of the Li molar fraction when the films are subsequently annealed. This new electrolyte-free process enables a delithiation of LCO up a Li molar fraction of *x* ≈ 0.5 without compromising the crystalline quality. Further delithiation is possible up to *x* ≈ 0.4 in the surface layer probed. The Li extraction process generates modifications in LCO electronic structure due to charge compensation. We describe the formation of Co^4+^ and identify the spectroscopic features of this ion, as evidenced by XPS and XAS results. We find a concomitant signal from holes located at O atoms after removing Li, which indicates that both Co and O atoms compensate the charge. We identify a (2 × 1) surface reconstruction related to a surface termination by 0.5 ML of Li. In situ delithiation of epitaxial films of LCO by this method can be used in future work to prepare solid-state interfaces of high crystalline quality and to investigate the electronic structure and the state of charge of electrodes, relevant information both for battery and for electrochemical random access memories (ECRAM) studies.

## Supporting Information Available

Detailed representation of crystal structure of LCO (SI.1); XPS overview of stoichiometric material (SI.2); simulations of the effect and depth of ion bombardment (SI.3); Li 1s XPS core level at different energies (SI.4); relative intensity evolution of components in the XPS Li 1s upon Li deintercalation (SI.5); comparison and deconvolution of Co 2p and Co 3p core-level spectra at different delithiation states (SI.6); scheme and LEED patterns describing possible surface reconstructions (SI.7); and valence band for stoichiometric LCO (SI.8) (PDF)
