# Supplementary material for: Physical Delithiation of Epitaxial LiCoO2 Battery Cathodes as a Platform for Surface Electronic Structure Investigation
Source: ACS Appl Mater Interfaces. 2023 Jul 19;15(30):36224–32. doi: 10.1021/acsami.3c06147 (PMC10401565; doi:10.1021/acsami.3c06147)
Supplement: Supplementary file 1 — am3c06147_si_001.pdf [file am3c06147_si_001.pdf]

Supporting Information

Physical delithiation of epitaxial LiCoO2 battery cathodes as a platform for surface electronic structure investigation

Elena Salagre[1], *Pilar Segovia*[1,2], *Miguel Angel González-Barrio*[3], *Matteo Jugovac*[4], *Paolo Moras*[4], *Igor Pis*[5], *Federica Bondino*[5], *Justin Pearson*[6], *Richmond Shiwei Wang*[6], *Ichiro Takeuchi*[6], *Elliot J. Fuller*[7], *Alec A. Talin*[7], *Arantzazu Mascaraque*[3] *and Enrique G.* Michel[1,2*]

1. Dto Física Materia Condensada, Univ. Autónoma de Madrid, Spain

2. IFIMAC (Condensed Matter Physics Center), Univ. Autónoma de Madrid, Spain

3. Dto. Física de Materiales, Fac. Ciencias Físicas, Univ. Complutense de Madrid, Spain

4. Istituto di Struttura della Materia-CNR (ISM-CNR), Trieste, Italy

5. IOM CNR Laboratorio TASC, AREA Science Park, Trieste, Italy

6. Materials Science and Engineering, Univ. of Maryland, College Park (MD), USA

7. Sandia National Laboratories, Livermore (CA), USA.

**Corresponding Author: enrique.garcia.michel@uam.es*

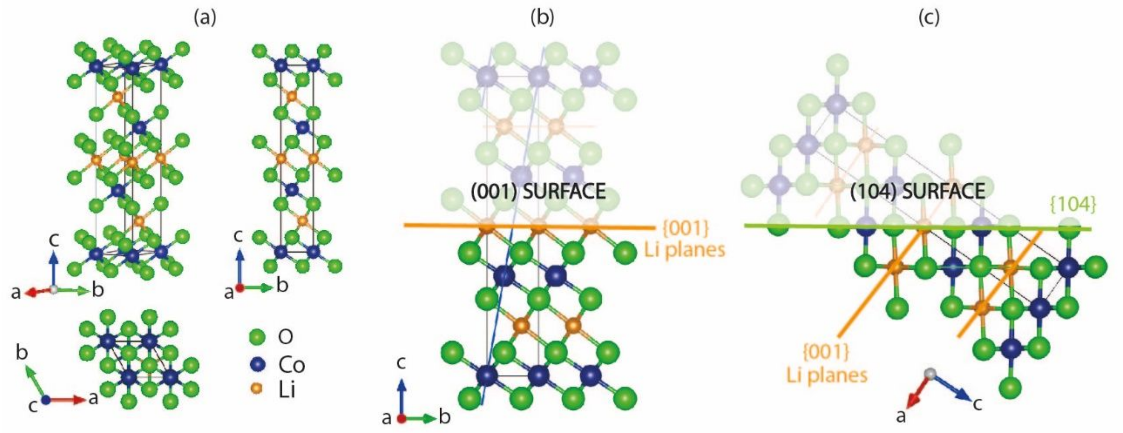

Figure SI 1: (a) $LiCoO_2$ crystalline structure in hexagonal axes for different views. (b) LCO (001) and (c) LCO (104) sample orientations highlighting the exposed crystalline planes in each case. A hexagonal conventional unit cell and axis are used. Colored circles are used to represent Co (blue), O (green), and Li (orange) atoms. Orange and blue lines represent Li and Co planes, respectively.

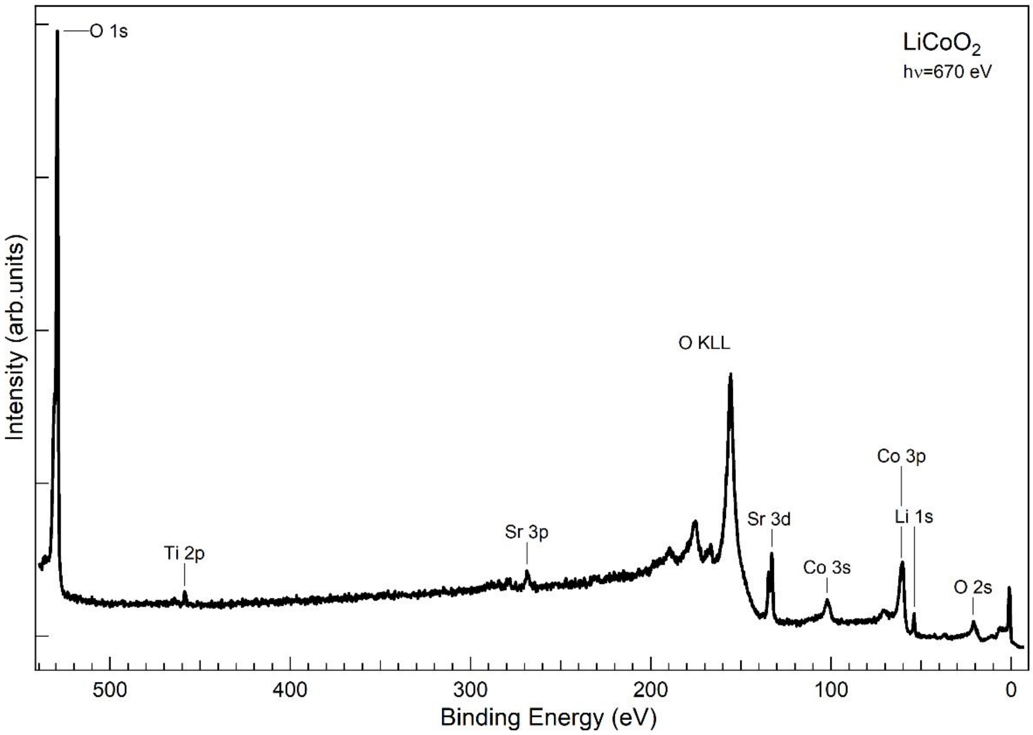

Figure SI 2: XPS overview taken with hν = 670 eV. The main core levels and Auger peaks are labelled. Note the absence of C 1s, evidencing a negligible surface contamination. Substrate peaks area visible.

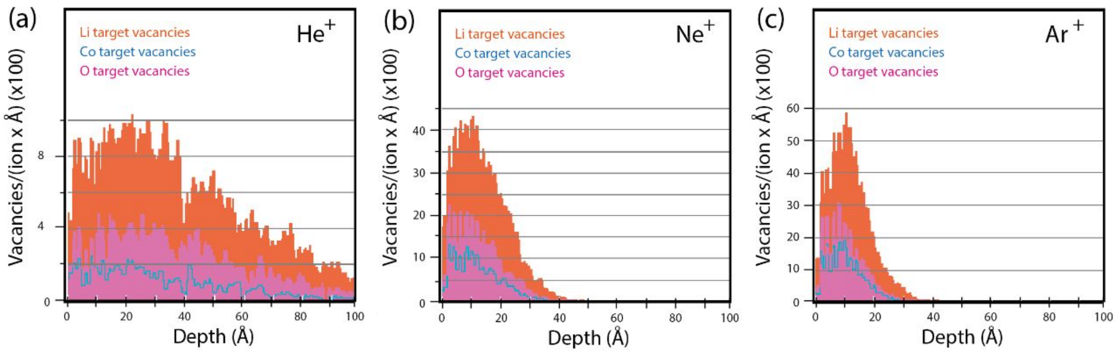

Figure SI 3: Results of TRIM simulations[1,2] for vacancies created by bombardment with 500 eV ions: Y axis represents vacancies per ion per Å. This number, multiplied by the ion dose (in ions/m^2) yields the vacancies per unit volume. (a) He^+ produces few vacancies, and the ions penetrate in the material more than 10 nm; (b) Ne^+ and (c) Ar^+ produce more vacancies, and the ion damage extends only to 5 nm. The Li/Co vacancies ratio is higher for Ne^+ than for Ar^+ (5 Li vacancies per Co vacancy for Ne^+ and only 3.3 for Ar^+). For this simulation we have used a standard value of about 25 eV for Co and O displacement energies. For the displacement energy of Li we have used 5 eV, a number more in accordance with the high diffusivity of Li in LCO.

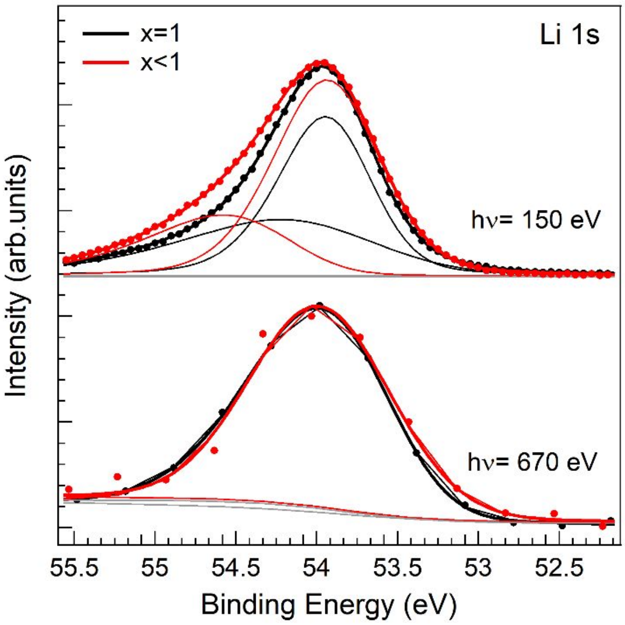

Figure SI 4: Li 1s core level taken at different photon energies highlights the dependency in the asymmetry of the peak with the photon energy, probing its surface character.

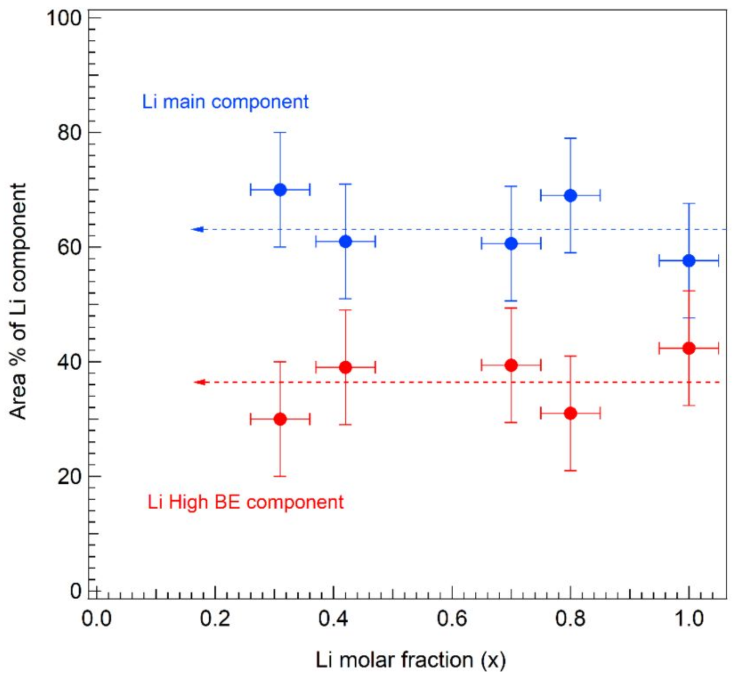

Figure SI 5: Relative intensity of the two components of Li 1s peak from Fig. 3 in main text as a function of Li molar fraction.

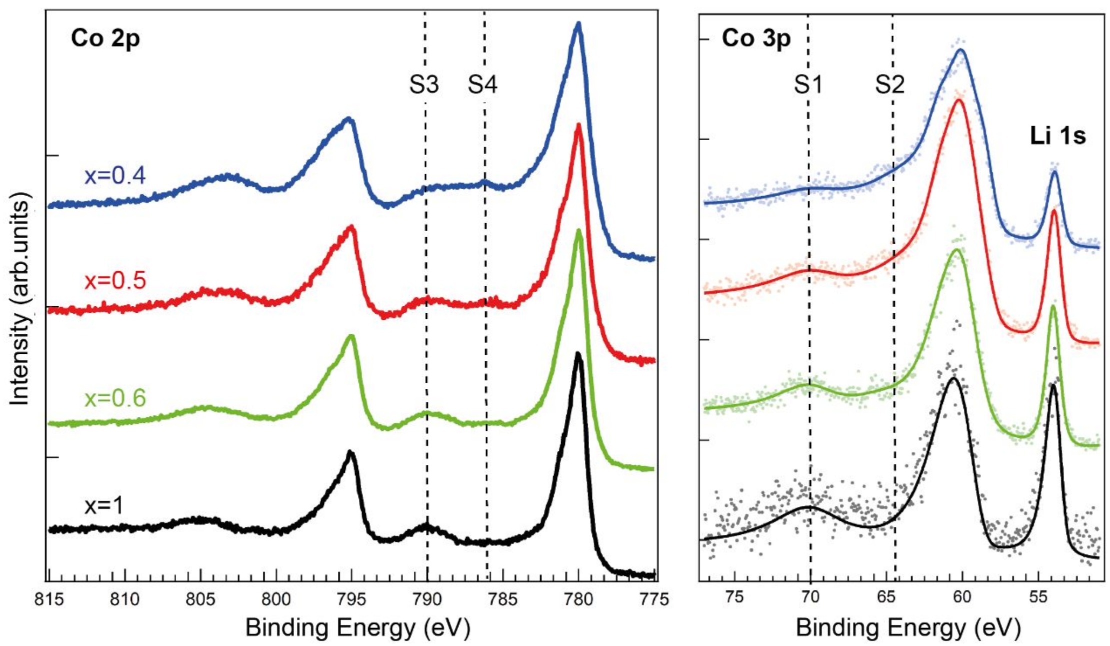

Figure SI 6: Co 2p and Co 3p spectra taken with hv=1000 eV and hv=300 eV respectively. Satellites S1 (for Co 3p) and S3 (for Co 2p) are typical of Co^{3+} and are seen with high intensity in the stoichiometric sample. Satellites S2 (for Co 3p) and S4 (for Co 2p) are typical of Co^{2+} and are observed only for high delithiation (low Li molar fractions) due to partial damage.

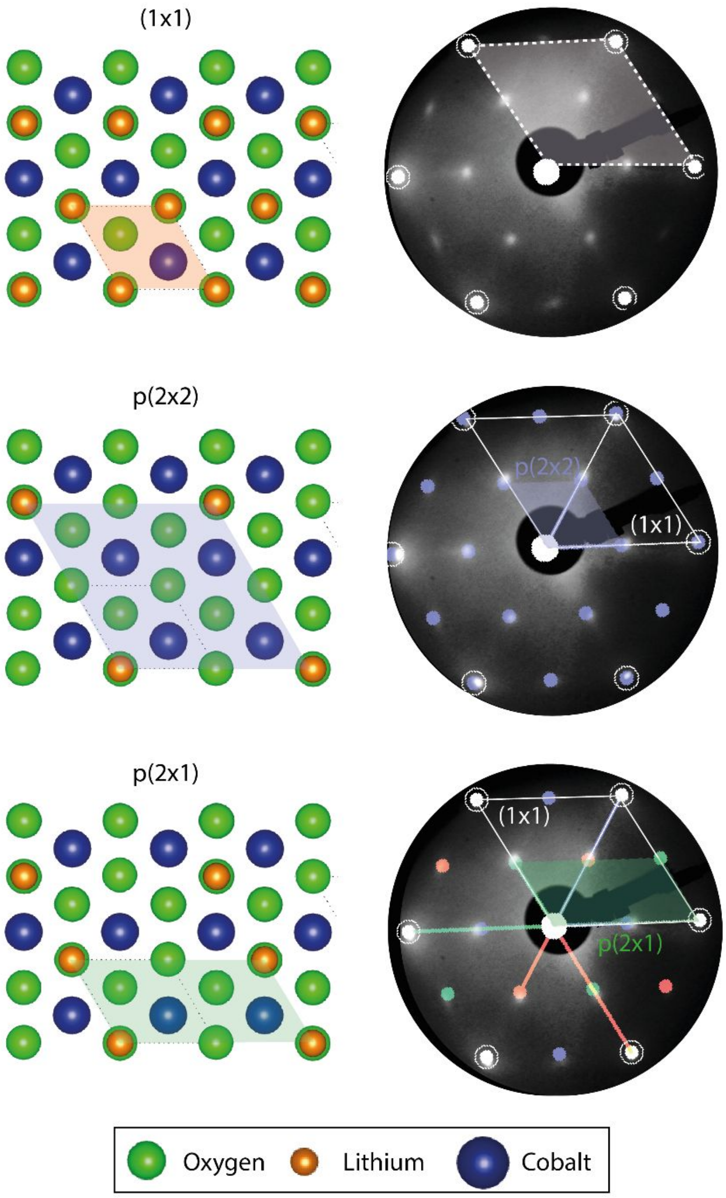

Figure SI 7: Left: Possible real space arrangement of a Li surface termination for a (1x1) (Li coverage: 1 ML), a (2x2) (Li coverage: 0.25 ML) and (2x1) (Li coverage: 0.5 ML) reconstructions. These idealized atomic arrangements are only simple models compatible with the LEED patterns observed and are not based on specific experimental evidence. Right: LEED patterns observed and scheme of the superstructures.

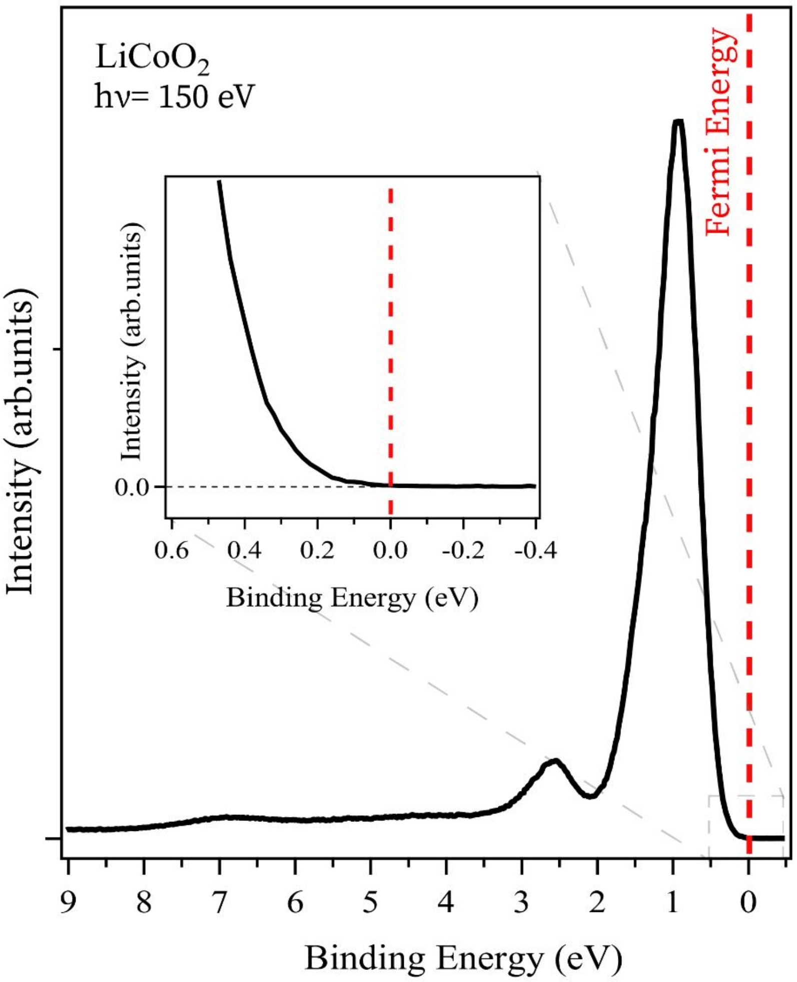

Figure SI 8: Integrated valence band of stoichiometric LCO. The dashed vertical line highlights the position of the Fermi energy and the insulating character of the sample. The inset shows an enlarged view of the region closer to the Fermi energy.

References:

1. Ziegler, J. F., Ziegler, M. D. & Biersack, J. P. SRIM – The stopping and range of ions in matter (2010). *Nucl Instrum Methods Phys Res B* **268**, 1818–1823 (2010).
2. Ziegler, J. F. SRIM-2003. *Nucl Instrum Methods Phys Res B* **219–220**, 1027–1036 (2004).
